# Optically Controllable Linear-Polarization Rotator Using Chiral-Azobenzene-Doped Liquid Crystals

**DOI:** 10.3390/ma10111299

**Published:** 2017-11-13

**Authors:** Cheng-Kai Liu, Chian-Yu Chiu, Stephen M. Morris, Min-Cheng Tsai, Chii-Chang Chen, Ko-Ting Cheng

**Affiliations:** 1 Department of Optics and Photonics, National Central University, Taoyuan 32001, Taiwan; aerobert007@gmail.com (C.-K.L.); abbbc987654321@gmail.com (C.-Y.C.); ecckk0201@gmail.com (M.C.T.); trich@dop.ncu.edu.tw (C.-C.C.); 2 Department of Engineering Science, University of Oxford, Oxford OX1 3PJ, UK; stephen.morris@eng.ox.ac.uk

**Keywords:** liquid crystal, polarization, chiral azobenzene

## Abstract

A linear-polarization rotator based on the optically tunable pitch of chiral-azobenzene-doped liquid crystals (CAdLCs) has been investigated. It is shown that the orientation of linearly polarized (LP) light can be optically tuned using CAdLCs and that the transmitted light possesses a good degree of linear polarization (DoLP). Experimental and simulation (4 × 4 Berreman matrix) results show that the rotation angle is dependent on the pitch as well as the number of turns of the cholesteric LC helix. Some causes to affect the DoLP of the output LP lights during photoisomerization are also discussed. Moreover, a calibration term, *β*(*t*), is also introduced to elucidate the behavior of the discontinuous change of the CAdLC pitch in a fixed cell thickness.

## 1. Introduction

Over many decades, scientists in both the academic and industrial sectors have studied and exploited the remarkable optical properties of chiral nematic liquid crystals (LCs) (historically known as cholesteric LCs (CLCs)), particularly the well-known phenomenon of selective Bragg reflection [1]. Selective reflection can be observed when the wavelength of the incident light is comparable to the pitch of the CLC [1,2]. The central reflection wavelength (*λ_o_*) defined by the Bragg condition can be described as

*λ_o_* = *nP*_0_cos*θ*, (1)

where *n*, *P*_0_, and *θ* are the average refractive index of the LCs, pitch, and the angle between the incident light and the helical axis of the CLC, respectively. The difference in the number of turns of the CLC helix between the two Grandjean disclination lines in a wedged cell is 0.5, indicating that the pitch can be automatically tuned to match the cell gap in accordance with the elastic free energy of the LC [3]. The number of turns of the CLC helix and the pitch will also be spontaneously adjusted to be a multiple value of one-half of the pitch (the period of the helical structure is actually one half of the pitch due to the n = −n invariance). Moreover, the pitch of a CLC in a glass cell has been shown to vary with temperature in a stepwise manner [4,5,6,7,8,9,10]. Because CLCs are typically sandwiched between two rigid glass substrates, a pitch jump in the CLCs ensures that the number of turns of the helix satisfies the one half-integer number required to fit within the cell gap. Moreover, Belyakov et al. and Zink et al. have both reported that the pitch variation is related to the surface anchoring energy [7,8,9,10].

The pitch of a photosensitive-material-doped CLC can also be modified using light illumination [11,12,13,14,15]. The key material for demonstrating an optically tunable wavelength-dependent linear-polarization rotator is the azo chiral dopant, also known as the chiral azobenzene dopant. Known for its phototunable chirality, this type of material has been studied extensively over the past decade. Considering chiral-azobenzene-doped LCs (CAdLCs), the central reflection wavelength and corresponding reflection band, which are dependent on the pitch of the CLC, can be optically tuned towards either red or blue wavelengths by a *trans*–*cis* photoisomerization process that is triggered by light illumination at a specific wavelength. Additionally, the *cis*-isomers also tend to naturally transform back to the stable *trans*-isomers over a certain period of time. The required time for naturally transforming azobenzenes from *cis*-isomers back to *trans*-isomers via dark relaxation is typically of the order of several tens of hours [11,12,13,14,15].

In addition to the well-known Bragg reflection, the application of CLCs, which can be used to control the polarization direction of linearly polarized (LP) light, has not been widely reported. Zhang et al. have successfully demonstrated a high-performance wedge-shaped CLC depolarizer [16]. They stated that the macroscopic spot of the light beam impinging on the CLC can be divided into a summation of micron-sized spots. Each micron-sized spot of the light beam passes through a specific position of the wedged-shaped CLC depolarizer, corresponding to a specific pitch of the CLC. Therefore, the polarization state and orientation of the linear polarization of each micron-sized spot can be modulated differently by the wedge-shaped CLC depolarizer. As a result, the output depolarized light beam is a combination of the micron-sized spots that make up the light beam with various orientations of the linear polarization. Their results indicated that CLCs can be used to control the polarization orientation of the outgoing beam. Nevertheless, the control of polarization rotation of transmitted LP light using phototunable CAdLCs has not been considered in detail.

Several techniques have been adopted to control the polarization orientation of LP light over the past few decades. Briefly, the polarization direction of LP light propagating through an optical device can be changed. One simple approach that can be employed to design a polarization rotator for LP incident light involves the combination of a polarizer and a half- or quarter-wave retardation plate [1,17,18]. However, the drawbacks associated with using such optical devices include the wavelength dependence, requirements of precise alignment and orientation of the optical components, and sensitivity to incident angles. To avoid the effect of a strong wavelength dependence of the incident light on the linear-polarization rotator, several precisely designed birefringence quarter-wave plates are required [19]. The fabrication processes become complicated, and the cost is therefore relatively high. Moreover, Fresnel rhombs are usually applied as an achromatic 90° polarization rotator based on total internal reflection. However, the dimensions of rhomb systems that are typically used are excessively large, which precludes them from being used in compact electronics applications because the propagation of the beam through the Fresnel rhombs generates a lateral shift caused by the refraction of light [18]. Twisted nematic LCs (TN-LCs) can also rotate the polarization of LP light within a certain limit. For a TN-LC that has a degree of twist characterized by *ϕ*, and a combination of parameters including the thickness (d), birefringence (∆n) of the LC, and wavelength (*λ*) of the incident light, which satisfy the Mauguin condition (i.e., *ϕ* << 2*π*dΔn/*λ*), the effect of polarization rotation effect is valid. If the incident LP light possesses a wavelength that satisfies the Mauguin condition, then the input linear polarization can be rotated by *ϕ* degrees upon passing through a TN-LC. Although the fabrication of TN-LCs is relatively simple compared with the preceding two techniques, the rotation angle *ϕ*, of an incident monochromic light source cannot be readily electrically/optically tuned in-situ continuously using a single device. However, the combination of the TN-LC and a Fabry–Perot resonator has been shown to function as a spectrally-selective linear polarizer [20].

In this work, an optically controllable linear-polarization rotator based on a CAdLC is reported. Experimental and simulation results reveal that CAdLCs can be employed to rotate the polarization of incident LP light from one orientation to another. It is found that the orientation of the polarization is dependent on both the pitch and the number of turns of the CLC helix. If the pitch can be tuned, then the rotation of the polarization of incident light can be controlled.

## 2. Materials and Methods

Two LC mixtures, namely, A and B, whose Bragg reflection bands were in the infrared (IR) region, were prepared as follows. LC mixture A was prepared by mixing 86.6 wt% of the nematic LC (726200-000, Fusol Material, Tainan, Taiwan) with 13.4 wt% of the right-handed chiral dopant (CB15, helical twisting power (HTP) ~7 μm^−1^ for 726200-000 at 25 °C, Fusol Material, Tainan, Taiwan). The pitch was calculated to be approximately 1066 nm. The refractive indices *n_e_* and *n_o_* of the LCs are 1.640 and 1.487, respectively, measured at a wavelength of 632.8 nm at 25 °C. LC mixture B, on the other hand, was prepared by mixing 97.3 wt% of the nematic LC (E7, Merck, Taoyuan, Taiwan) with 2.7 wt% of the left-handed chiral azobenzene dopant of Ql-3c-S. The initial pitches were calculated to be approximately 600 nm from the reflection bands. LC cells with gaps of ~15 µm, defined by spacer beads, were prepared by assembling two glass substrates coated with indium tin oxide and treated with homogeneous anti-parallel rubbed alignment layers. Finally, the homogeneously mixed LC mixtures A and B were then injected into the empty glass cells to produce the LC films. The edges of the LC cells were then sealed with epoxy.

## 3. Discussions

### 3.1. Effect of Changing the Number of Turns of the Helix on the Polarization Rotation Characteristics of CLCDs

**Figure 1:**
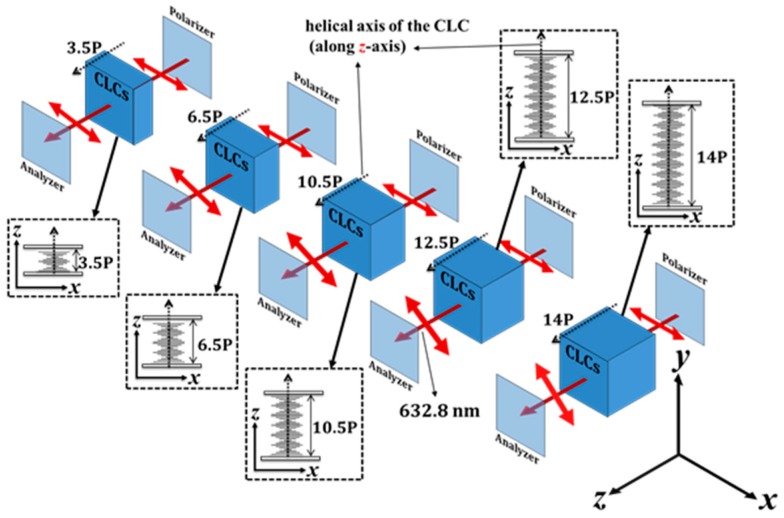
Experimental setup to investigate the rotation angle of LP (linearly polarized) light after passing through the CLC. The orientation of the linear-polarization of the transmitted beam is rotated in the *x*–*y* plane. P is the pitch of the five CLCs (cholesteric liquid crystals), whose helical axes are aligned along the *z*-axis. The five inserts in the dashed square represent the cross-sectional views of the five CLCs through the *z*–*x* plane.

Before demonstrating the ability to rotate the linear-polarization using CAdLC, some of the basic properties of the linear-polarization rotator using CLCs are introduced to aid the following discussions. Firstly, CLC mixture A was used to demonstrate the basic properties of the linear-polarization rotator based on a CLC. An He-Ne laser (*λ* = 632.8 nm) was selected to be the incident light source. A schematic of the experimental arrangement is shown in Figure 1. Various cell gaps (which in turn influence the number of turns of the CLC helix) were prepared to demonstrate the performance of the linear-polarization rotators. The numbers of turns of the helix of CLC mixture A (pitch ~ 1066 nm), filled into five different LC cells with cell gaps of 3.58, 6.95, 11.14, 13.38, and 15 µm, were calculated to be 3.5, 6.5, 10.5, 12.5, and 14, respectively. The calculations of the number of turns of the helix were based on the fact that the pitch will be automatically tuned to fit the cell gap. As a result, the number of turns will be spontaneously adjusted to be an integer multiple of 0.5. Thus, the calculated pitch lengths were 1023, 1069, 1061, 1070, and 1071 nm in the LC cells for cell gaps of 3.58, 6.95, 11.14, 13.38, and 15 µm, respectively. The differences in the pitch lengths were found to be small. Thereafter, each CLC linear-polarization rotator was placed between a linear polarizer and a linear analyzer to measure the rotation angle of the output He-Ne laser beam by the CLC linear-polarization rotators with various cell gaps. The angle between the transmission axis of the polarizer, which was used to linearly polarize the He-Ne laser, and the rubbing direction of the CAdLCs cell was set at 45° in order to achieve as close to a perfect polarization rotation as possible. The rotation angle of the outgoing LP light can be determined by rotating the transmission axis of the analyzer. To further check the above experimental results, the effect of the linear-polarization rotator was analyzed using simulations carried out using the 4 × 4 Berreman approach, which is described in detail elsewhere [21,22]. All the parameters for the simulation, such as the refractive indices of the LC, cell gap, CLC pitch, and transmission axes of the polarizer and analyzer, were identical to those described in the experimental part. However, the precise cell gap and the temperature and wavelength-dependent refractive indices of the LCs are extremely difficult to be precisely determined.

**Figure 2:**
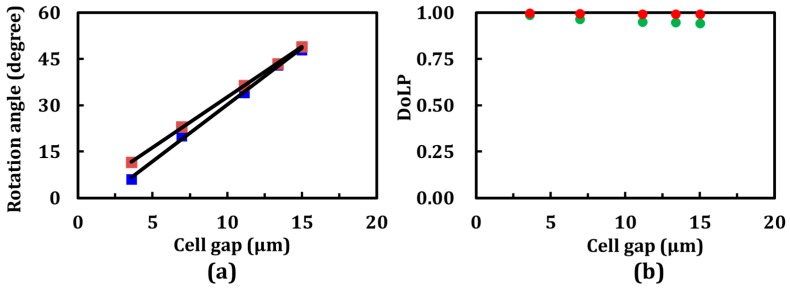
(**a**) Experimental and simulation results of the rotation angle as a function of the cell gap; (**b**) the DoLP (degree of linear polarization) values of the experimental results, shown in (**a**), as a function of the cell gap.

Experimental and simulation results reveal the different rotation angles of the LP He-Ne laser beam through the five CLC cells with the same pitch but different cell gaps, as shown in Figure 2a. Interestingly, the rotation angles for the CLC linear-polarization rotators were found to be linearly proportional to the number of turns of the CLC helix (cell gaps). However, comparing the experimental and simulation results, the rotation angles by these five linear-polarization rotators did not coincide with each other because of certain discrepancies in the physical parameters, including the cell gap and the temperature- and wavelength-dependent refractive indices of the LCs employed in this study. In addition, the planar alignment layers onto the substrates of the LC cells were nonuniform; as a result, the CLCs were not aligned in perfectly Grandjean textures. To evaluate the linearity of the output polarization, the degree of linear polarization (DoLP) is defined as the following:

(2) $$DoLP=\frac{\left(I_{\max}-I_{\min}\right)}{\left(I_{\max}+I_{\min}\right)},$$

where *I*_max_ and *I*_min_ are the measured maximum and minimum intensities of the output beam through a rotating polarizer, respectively. Figure 2b shows the DoLP values obtained from the experimental (green circles) and simulation (red circles) results, shown in Figure 2a, as a function of the cell gap. All values are found to be close to unity. These findings indicate that the transmitted beam was linearly polarized, and that the DoLP value was almost independent of the cell gap.

### 3.2. Effect of the Pitch on the Polarization Rotation Characteristics of CLCs

**Figure 3:**
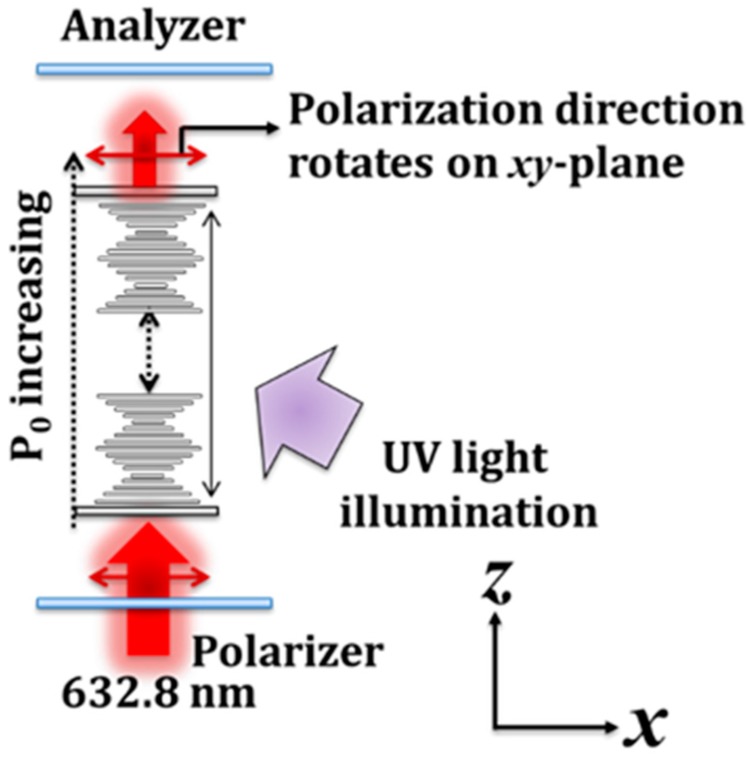
Experimental setup to investigate the rotation angle of the linear polarization of a He–Ne laser during UV illumination. The orientation of linear-polarization of the transmitted beam can be rotated in the *x*–*y* plane.

To control the rotation of the linear polarization, the optically active material, that is, the chiral azobenzene, was employed to demonstrate an optically controllable and wavelength-dependent linear-polarization rotator based on CAdLCs. LC mixture B was filled into a 15 µm-thick LC cell and was used to demonstrate such an optically controllable linear-polarization rotator. The experimental setup is illustrated in Figure 3. A LP He-Ne laser was used as the incident light source with which to examine the optically controllable linear polarization rotator when exposed to ultraviolet (UV) illumination. The angle between the transmission axis of the polarizer, which was used to define the state of linear polarization of the He-Ne laser, and the rubbing direction of the CAdLC cell was set at 45°. The CAdLCs were illuminated with a UV light source with a central wavelength of 365 nm and an intensity of 0.6 mW/cm^2^ so as to gradually increase the pitch via photoisomerization.

**Figure 4:**
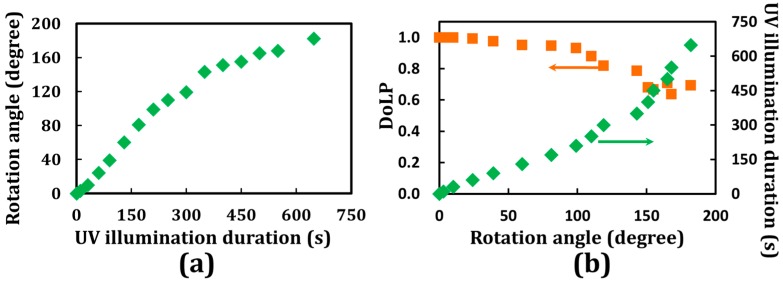
(**a**) Experimental results of the rotation angle as a function of the UV illumination duration; (**b**) experimental results of the DoLP as a function of rotation angle.

Experimental results indicated that the rotation angle of the incident LP probe beam increases with the duration of UV illumination, as shown in Figure 4a. The rotation angle of the output LP light ranging from 0° to ~180° can be controlled optically. To further understand the results of the optically controllable linear-polarization rotators, a cell gap parameter (*α*) after UV light illumination for a specific duration is introduced:

(3) α≡k(t)[P(t)+β(t)],

where *k*(*t*) and *P*(*t*) are the number of turns of the helix and the pitch of the CAdLC, respectively. *β*(*t*) is a calibration term, which is dependent on the UV illumination duration. The variable *t* represents the UV illumination duration. Additionally, *k*(*t*) equals 0.5*e*, where *e* is a positive integer (i.e., 1, 2, 3…) [3]. The cell gap parameter (*α*) and the pitch are 15 µm and 600 nm, respectively; hence, *k*(*t*) equals 25. Following photoisomerization of the doped chiral azobenzene molecules, the pitch gradually elongates with an increase in the UV illumination duration within a specific time limit. However, as the pitch is tuned from 600 nm to 610 nm (590 nm), if the number of turns of the CLC helix is kept constant (e.g., 30), the cell gap should be extended (shrunk) to 15.25 (14.75) µm. The preceding circumstance is unreasonable because the cell gap of CAdLC cell is invariable. Accordingly, the calibration term *β*(*t*) is added to calibrate *P*(*t*) to satisfy the condition of Equation (3). In other words, the calibration term indicates that there is a force, resulting from the alignment layers onto the substrates, that compresses or extends the pitch of a CLC as the pitch of a CLC increases or decreases during illumination in order to satisfy Equation (3). For a given cell gap of 15 µm, the *k*(*t*) or *P*(*t*) terms gradually decrease or increase with the duration of UV illumination because of the effect of photoisomerization. Based on Equation (3), considering three different UV illumination durations, that is, 2, 30, and 60 s, the cell gap parameter and the relationships of *k*(*t*), *P*(*t*), and *β*(*t*) can be expressed as the relationship *α* ≡ *k*(*2*)[*P*(*2*) + *β*(*2*)] = *k*(*30*)[*P*(*30*) + *β*(*30*)] = *k*(*60*)[*P*(*60*) + *β*(*60*)] = 15 μm, as shown in Table 1. The values of *k*(*t*) in order of increasing *t* are *k*(*2*) > *k*(*30*) > *k*(*60*). As shown in Figure 4a (experimental results), the rotation angles, *ϕ*(*t*), are arranged in order of increasing *t* are *ϕ*(*2*) < *ϕ*(*30*) < *ϕ*(*60*). Table 2 summarizes the relationship between *ϕ*(*t*), *k*(*t*), and *P*(*t*) as *t* = 2, 30, and 60 s based on the results shown in Figure 4.

**Figure 5:**
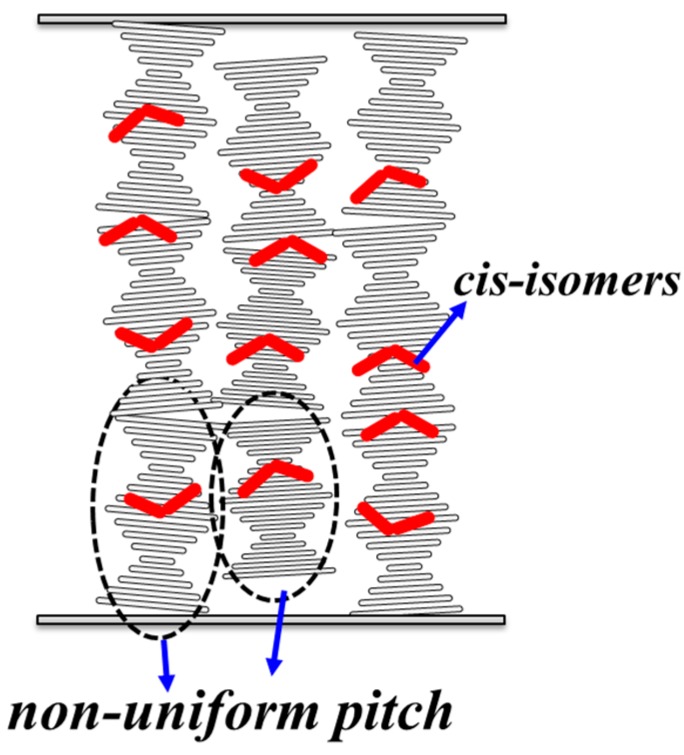
Schematic diagram of CAdLCs (chiral-azobenzene-doped liquid crystals) with a planar texture (helix axis normal to the substrates) that were disturbed by the bent-shaped *cis*-isomers after UV illumination for a long period of time. Moreover, the local pitch could be nonuniform due to variations in the cell thickness and nonuniform alignment layers.

Considering a CAdLC cell with a fixed cell gap, the rotation angle increases with the decreases [increases] of *k*(*t*) [*P*(*t*)]. Usually, the *P*(*t*) cannot be unlimitedly increased, so the valid CLC pitch for the polarization rotator will be discussed later [14]. The results obtained for the DoLP are shown in Figure 4b. The values of DoLP are found to be very close to unity when the rotation angle is smaller than 50°. This finding indicates that the polarization of the outgoing light is a near-perfect LP light beam. However, the DoLP value decreases as the rotation angle increases. A larger rotation angle is obtained for a longer pitch by exposing the cell to UV illumination for a longer period than usual. Therefore, more *cis*-isomers are generated and consequently disturb the planar texture and the pitch of the CLCs. Figure 5 depicts the planar textures of CAdLCs that are disturbed by the bent-shaped *cis*-isomers [23,24,25,26]. This drawback can be overcome if the materials of chiral azobenzene with high HTP can be obtained. Briefly, to obtain CLCs with a specific pitch, the required concentration of the chiral dopant should be decreased with the increase in the HTP value of the selected chiral materials. Hence, to approach the same pitch of CLCs, (600 nm), the required concentration of the chiral azobenzene with high HTP can be reduced to decrease the population of *cis*-isomers that disturb the uniformity of planar texture and pitch after photoisomerization (from *trans*- to *cis*-isomers). Thus, the uniformities of CLC structures and the rotation angle of the output LP light can be improved significantly. Another method to increase the DoLP is to keep the orientation of the CLC in a planar-aligned (Grandjean) texture by employing an LC with a negative dielectric anisotropy and applying an external electric field [27]. Figure 5 shows that the local pitch of the CLCs in the bulk of the LC cell after long UV illumination could be nonuniform due to the various photoisomerization rates of azobenzenes in different regions, caused by cell fabrication errors, such as inhomogeneous alignment layer and nonuniform cell gap [1,3]. The probe beam passed through the area of the CAdLCs, where the local pitch was nonuniform caused by the cell gap fabrication errors.

**Figure 6:**
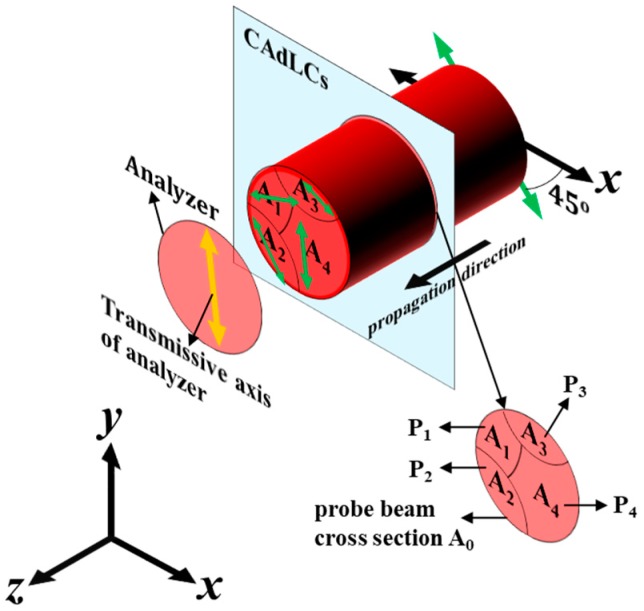
Illustration of the combined linear polarizations of the output beam through the cross-section of CAdLCs where the local pitch is nonuniform.

Figure 6 shows a schematic diagram of the cross-section of a CAdLC cell for an incident probe beam propagating along *z*-axis through mixture B (Figure 4) following UV illumination for a specific duration. The angle between the polarization direction of the incident LP probe beam and the *x*-axis is set to be 45°. We assume that the pitches in these four different subareas, namely, A_1_, A_2_, A_3_, and A_4_, are different due to the cell fabrication errors after a certain UV illumination duration. The number of the generated subareas could be more or less than four (Figure 6), depending on how high the cell gap fabrication errors are. The probe beam goes through subareas A_1_, A_2_, A_3_, and A_4_, and their various pitches are P_1_, P_2_, P_3_, and P_4_, respectively. We assume that the pitch of mixture B (Figure 4) can be supposedly tuned from the initial pitch (600 nm) to the expected pitch, P_4_, after a certain UV illumination duration, and the P_1_, P_2_ and P_3_ are those nonuniform pitches caused by the cell gap fabrication errors, such as the variations in the cell thickness and the nonuniform alignment layers. Hence, these four pitches are different. Based on the results shown in Figure 4b, the linear polarization directions (rotation angles), denoted by the four green double-head arrows in Figure 6, of the four output LP lights passing through the four subareas are different. Accordingly, as the transmission axis of the analyzer rotates to be parallel to the linear polarization of output LP light, passing through subarea A_4_, the DoLP of the output LP light passing through the whole cross-section decreases with the increase of the UV illumination duration because the polarization direction of the output LP light is the sum of the four output LP lights. Hence, the calculated DoLP value of the output light decreased because of the combined LP light, but not the elliptically polarized output light [28].

**Figure 7:**
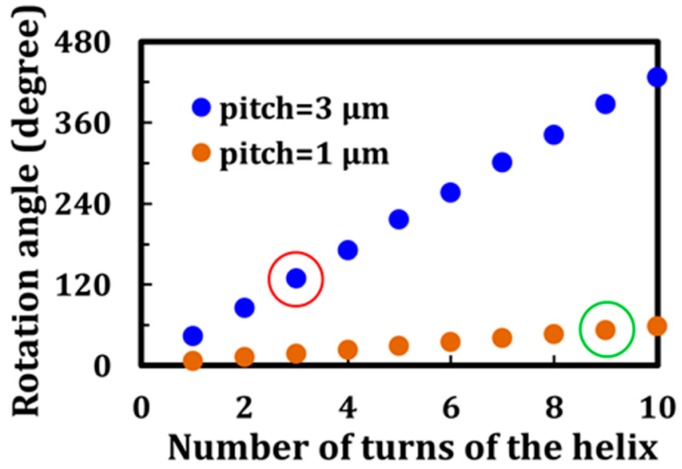
Simulation results for the rotation angle of the polarization as a function of the number of turns of the helix confined within a glass cell.

Simulations using the 4 × 4 Berreman matrix help to investigate the effect of the CLC pitch on the rotation angle. Figure 7 shows the simulation results for the rotation angle in terms of the pitch and number of turns of the CLC helix. The wavelength of the incident LP light and the LC material used in this study were 632.8 nm and E7, respectively. For the same number of turns of the helix, the rotation angle for LP red light by a CLC with a pitch of 3 μm is found to be larger than that obtained for a sample with a pitch of 1 μm. Moreover, regarding the same cell gap (9 µm), the rotation angle for LP red light by a CLC cell with a pitch of 3 μm and 3 turns of the helix (red circle in Figure 7) is found to be larger than that by a CLC cell with a pitch of 1 μm and nine turns of the helix (green circle in Figure 7). Table 3 shows the DoLP values of all the plotted data, depicted in Figure 7, based on the simulations. All of the DoLP values are close to unity, indicating that the output light of the plotted data is LP.

Based on the simulated results shown in Figure 2b and Table 3, the DoLP values of the output light are very close to unity. Therefore, for a homogeneous LC cell with a given thickness, the CLC pitch for the polarization rotator is smaller than the LC cell gap. As the pitches are longer than the cell gap, the CLC cells could become 90° TN-LCs or 180° super TN-LCs [1]. The limitation of pitch can be understood because of setting the angle between the polarization direction of the incident light, and the rubbing direction of the CAdLC cell was 45°. The DoLP of the output LP light after passing through the LC cell could not be good enough. Moreover, for cells with a typical thickness of 3 µm and original pitch of 600 nm (Figure 4), the pitch should be tuned to 12 µm (6 µm) using photoisomerization to form 90° TN-LCs (180° super TN-LCs). Hence, such a chiral azobenzene is difficult to prepare for real applications [14].

## 4. Conclusions

Polarization rotation and optically-induced tunable pitch of CLC for linear polarization rotation have been investigated. With an electrically controllable pitch of CLCs as reported by Xiang et al. and Xianyu et al. [29,30], a CLC polarization rotator can be utilized in various applications, such as lasers, displays, communications, etc. In the past few decades, the research scope of CLCs has been mainly limited to the category of displays, light shutters, lasers, and smart windows [1,2,4,5,12,13,14,15,21,26,31,32,33,34]. The authors hope that the reported investigations and analysis can further extend to other fields, especially for the ability to rotate the polarization orientation using external stimuli. Most importantly, in this paper, we have reported and demonstrated an optically controllable linear polarization rotator experimentally, which is supported by results obtained using the 4 × 4 Berreman matrix. A phenomenological description of the polarization rotation is introduced that is based on the pitch and number of turns of the CLC helix, and the angle of 45° between the transmission axes of the polarizer. These were developed in accordance with the modeling of the reflection of cholesteric LCs using the Jones matrix, proposed by Yang et al. [35]. The detailed theory of calibration term, *β*(*t*), is also under development. Moreover, to overcome the reduction of the DoLP after the long exposure to UV illumination (see Figure 4b), chiral azobenzenes with very high HTP would be preferred to reduce the disturbance introduced from the bent-shaped *cis*-isomers [14].

## Figures and Tables

**Table 1:** Relationship of *α*, *β*(*t*), *k*(*t*), and *P*(*t*) as *t* = 2, 30, and 60 s and *α* = 15 µm.

UV Illumination Duration, *t*	Cell Gap Parameter, *α*
*t* = 2 (s)	*α = k*(*2*)[*P*(*2*) + *β*(*2*)] = 15 μm
*t* = 30 (s)	*α = k*(*30*)[*P*(*30*) + *β*(*30*)] = 15 μm
*t* = 60 (s)	*α= k*(*60*)[*P*(*60*) + *β*(*60*)] = 15 μm

**Table 2:** Relationship of *ϕ*(*t*), *k*(*t*), and *P*(*t*) as *t* = 2, 30, and 60 s.

UV Illumination Duration, *t*	Relationship of (*t*), *k*(*t*), and *P*(*t*)	Description
*t = 2*, *30*, and *60* (*s*)	*ϕ*(*60*) > *ϕ*(*30*) > *ϕ*(*2*)	*ϕ*(*t*) increases with time, *t*
	*k*(*60*) *< k*(*30*) *< k*(*2*)	*k*(*t*) decreases with time, *t*
	*P*(*60*) > *P*(*30*) > *P*(*2*)	*P*(*t*) increases with time, *t*

**Table 3:** DoLP (degree of linear polarization) values for all the data shown in Figure 7.

**Pitch of 1 μm**										
**Number of Turns of the Helix**	1	2	3	4	5	6	7	8	9	10
**DoLP**	0.999	0.997	0.995	0.993	0.990	0.988	0.987	0.987	0.987	0.988
**Pitch of 3 μm**										
**Number of Turns of the Helix**	1	2	3	4	5	6	7	8	9	10
**DoLP**	0.883	0.997	0.904	0.989	0.908	0.975	0.923	0.968	0.925	0.949
